# Combined Topical Growth Factor and Protease Inhibitor in Chronic Wound Healing: Protocol for a Randomized Controlled Proof-of-Concept Study

**DOI:** 10.2196/resprot.8327

**Published:** 2018-04-27

**Authors:** Michael Stacey

**Affiliations:** ^1^ Division of Vascular Surgery Department of Surgery McMaster University Hamilton, ON Canada

**Keywords:** leg ulcers, growth factors, protease inhibitors

## Abstract

**Background:**

Leg ulcers due to venous disease are chronic wounds that can take 6 or more months to heal. Growth factors have been used to try and improve this healing; however, many such studies have failed, and that is thought to be due to enzymes in the wound that degrade the growth factors and prevent them from working.

**Objective:**

This is a proof-of-concept study that will evaluate the treatment of chronic leg ulcers with topically applied growth factors that are combined with a therapy to prevent their inactivation in the wound. This combined therapy has the potential to speed up the healing of these wounds and thereby improve the quality of life of patients and reduce the costs to the health system.

**Methods:**

This will be a double-blind, placebo-controlled, randomized controlled proof-of-concept study comparing growth factor with protease inhibitor wound dressings to growth factors with standard wound dressings.

**Results:**

The project was funded by the Canadian Institutes for Health Research and enrollment is expected to be initiated in 2018. It is expected that results will be available in 2021.

**Conclusions:**

It is expected that the results of this trial will inform as to whether modifying the wound environment through the use of protease inhibitors increases the effectiveness of topically applied growth factors in the healing of chronic wounds.

**Trial Registration:**

ClinicalTrials.gov NCT02845466; https://clinicaltrials.gov/ct2/show/NCT02845466 (Archived by WebCite at http://www.webcitation.org/6yOPhSBUA)

## Introduction

### Chronic Wounds

Chronic wounds are prevalent across the Canadian health care continuum [1] and have a significant financial and social burden on the health care systems, patients, and their families [1-4]. Chronic wounds are defined as wounds that are poorly healing and persisting for 3 months or more [1]. Clinical subtypes of chronic wounds include the following: (1) venous leg ulcers (VLUs), (2) arterial ulcers, (3) diabetic foot ulcers (DFUs), and (4) pressure ulcers. VLUs and DFUs are the most prevalent chronic wounds and have high rates of recurrence ranging from 16% at 1 year to 60% at 5 years for VLU [5,6] and greater than 50% after 3 years for DFU [2]. The cost attributed to chronic wound management in Canada is considerable [7] with more than Can $1 million per year estimated for each Canadian hospital to treat just pressure ulcers and surgical wound infections [8]. The total annual cost associated with DFU-related care was $547 million CAD, or $21,371 CAD annual cost per prevalent case in 2011 [3]. It is imperative that new cost-effective treatments that significantly increase healing rates and reduce amputation and hospital admission rates are required. Cost-effective wound management is a key priority for Canadian health care organizations [9-11].

### Venous Leg Ulcers

Lower limb ulcers occur in 1% to 2% of adults, with 70% of leg ulcers being of venous etiology [12]. The point prevalence in the population over 70 years of age is approximately 1% [13]. The majority of VLUs are long-standing; 60% of ulcer patients in the community experience ulcers that remain unhealed for more than 26 weeks, and 23% for more than 2 years [14]. In their Canadian Bandaging Trial, Harrison and colleagues identified that most patients were over 65 years in age and over two-thirds had experienced leg ulcers for many months. Half of the affected population had a leg ulcer history spanning from 5 to 10 years and one-third exceeding 10 years [15]. Some ulcers require skin grafting to achieve healing. The delayed healing of leg ulcers and the high costs associated with outpatient, inpatient, and community care represent a significant economic burden to the health care system, estimated to be in the order of 1% of total health care budget in Western societies [16]. Current clinical treatment guidelines for venous ulcers involve the use of compression bandaging to counter the effects of venous hypertension and dressings to promote an optimum wound-healing environment. Despite treatment with compression, healing times are still long, with only 45% of ulcers healing in 13 weeks [17]. There is a need for cost-effective therapies that improve these ulcer-healing rates.

### Diabetic Foot Ulcers

Diabetes is one of the most burdensome chronic diseases in Canada with an estimated 7% of the population living with diabetes [17]. Over their lifetime, 15% to 25% of people with diabetes will develop a DFU [18], and 12% of those patients will require lower extremity amputation due to the progression of their chronic DFU [19]. The current standard for treating DFUs is pressure off-loading in addition to treatments for wound infection, maintaining adequate blood supply, local wound care, and control of the diabetes. There is a need for cost-effective therapies that improve ulcer-healing rates and prevent amputations.

### Impaired Healing in Chronic Wounds

Wound healing is a complex process that has 4 phases [20]. The initial hemostatic phase occurs immediately after the injury, and during this phase, growth factors released from platelets that bind to the wound site initiate the wound-healing process. Growth factors are soluble signaling proteins that instruct cells during cell development and influence the process of wound healing [21]. The second phase is the inflammatory phase during which white cells migrate to the wound and release additional growth factors as well as remove foreign material. The third phase is the proliferative phase, which involves formation of granulation tissue, wound matrix formation, and epithelialization. The final phase is remodeling, which is an ongoing phase during which excessive material is removed and collagen synthesis and breakdown occur as the wound is remodeled. The last 3 phases overlap over time.

Chronic wounds are typically characterized by an ongoing and excessive inflammatory phase while proliferative phase is trying to occur at the same time [22]. This profound inflammatory phase is thought to play a key role in delaying healing. During this phase, there is evidence of elevated levels of inflammatory cytokines [23-25], elevated levels of proteases including matrix metalloproteases (MMPs) and elastase [22,26-28], impaired function of cellular components including fibroblasts and epithelial cells [29-31], and reduced levels of growth factors [22,32,33]. Contaminating bacteria also affect the inflammatory phase [34-36]. In chronic wounds, impaired healing is considered to be caused by a number of factors that are a result of this prolonged inflammatory phase: (1) degradation of extracellular matrix (ECM) proteins that form the new healed tissue, (2) degradation of healing regulators including peptide growth factors and endogenous protease inhibitors [22,27,28,37], and (3) impaired responses of the cellular components within the wounds [30,31,38].

### Increased Protease Activity in Wounds and Wound Fluid

The levels of a number of proteases and their inhibitors have been measured in different chronic wound types. These have assessed levels of proteases within chronic wound fluid (CWF), have compared levels between acute and chronic wounds, and have compared levels between nonhealing and healing chronic wounds. Results indicate that CWF has consistently elevated levels of MMPs (MMP-1,-2,-8,-9,-13) and neutrophil elastase compared with acute wound fluid [22,26,28]. Studies have demonstrated a reduction in MMPs during healing phases of VLUs and DFUs and reduced levels of tissue inhibitors of metalloproteinases (TIMPs) in nonhealing or reduced MMP/TIMP ratios during healing [23,37]. Although the levels vary between individuals, these studies indicate an association between lower levels of MMPs and healing of VLUs and DFUs.

In addition, the elevated levels of proteases within CWF are inhibitory to the healing process within chronic wounds by degrading ECM proteins and regulators of the healing process. Therefore, reduction of protease levels in CWF has the potential to improve the healing process in these wounds.

### Reduction in Protease Activity

A number of potential agents have been identified to reduce or modulate MMP activities and to control the proteolytic environment in the chronic wound. These include the antibiotic doxycycline [39], the wound dressing Promogran (Systagenix) [40], and nanocrystalline silver [41]. Promogran is a protease-modulating matrix wound dressing composed of 55% collagen and 44% oxidized regenerated cellulose in an absorbent open-pored, sterile, freeze-dried matrix. Once the matrix comes in contact with the wound exudate, it absorbs the fluid and forms a soft gel. The gel physically binds to and inactivates the damaging proteases (MMPs) and elastase and also binds to growth factors, protecting them and preventing them from being degraded [42]. The growth factors are then released back into the wound in an active form [40]. One small clinical study has shown a decrease in protease activity in CWF samples taken from patients with chronic venous ulcers treated with Promogran [43]. This study demonstrated a significant reduction of elastase and MMP-9 activity after 5 days of treatment with Promogran. Accelerated healing in both VLUs and DFUs has been observed in clinical trials with Promogran alone [44-46].

A small observational study of 15 patients indicated that Promogran, in combination with Regranex, had a stimulating effect on DFUs, with rapid wound area reduction [47]. Moreover, a small randomized prospective clinical study showed that the combination of Promogran and autologous growth factors from platelets was significantly better than the Promogran or autologous growth factors from platelets alone in DFUs [48]. Larger analyses do not show a significant benefit from applying topical autologous growth factors from platelets to wounds [49]. It is possible that the protease inhibition enabled the effect that was observed in this study.

### Enhancing Delayed Healing

Wound repair is controlled by a number of growth factors that include platelet-derived growth factor (PDGF), keratinocyte growth factor, transforming growth factor β, and a number of other growth factors. The increasing understanding of the role of growth factors, including PDGF and transforming growth factor-β, in wound repair has resulted in their clinical application for the treatment of chronic wounds. Over the past decade, there have been an increasing number of randomized controlled trials using PDGF in the format of becaplermin gel (Regranex). Becaplermin (recombinant human PDGF-BB, rhPDGF-BB) is a homodimer produced by recombinant DNA (deoxyribonucleic acid) technology utilizing the insertion of the gene for the B chain of PDGF into the yeast *Saccharomyces cerevisiae*. The biological activity of rhPDGF-BB is similar to the naturally occurring PDGF and includes formation of granulation tissue at the wound site and stimulation of the wound-healing processes [50].

There have been a number of trials for the treatment of nonhealing DFUs demonstrating statistically significant effects in phase 2, phase 3, and phase 4 clinical trials [51,52]. These trials have demonstrated the efficacy of rhPDGF-BB gel at 30 g/g and 100 g/g when used in conjunction with optimal wound care. Patients treated with rhPDGF-BB gel had a significant increase in complete healing compared with patients given placebo gel (50% vs 36% at dose of 100 g/g) and a decrease in the time to complete healing by 30% (14 weeks vs 20 weeks) [53]. It was concluded that rhPDGF-BB gel used in conjunction with optimal wound care is effective and well tolerated in patients with full-thickness DFUs. A systematic review and meta-analysis has concluded that rhPDGF-BB gel improves the healing of DFUs [54].

In other types of chronic wounds such as pressure ulcers, studies using rhPDGF-BB gel showed promising results [55,56]. In patients with chronic venous ulcers, 2 small randomized, placebo-controlled trials have evaluated the application of rhPDGF-BB gel (100 mg/g). In both studies, a greater percentage of patients treated with rhPDGF-BB gel achieved complete healing compared with patients in the placebo-treated group, but this was not statistically significant [57].

There is an ongoing safety review and warning from the US Food and Drug Administration regarding the amount of rhPDGF-BB gel that can be used. In a retrospective study of patients treated with rhPDGF-BB gel, the incidence rate for all cancers was 10.2 per 1000 compared with 9.2 per 1000 in matched controls [58]. An increased rate of mortality secondary to malignancy was observed in patients treated with 3 or more tubes of rhPDGF-gel in a postmarketing retrospective cohort study. A warning is now included in the product label, indicating the increased rate of mortality secondary to malignancy in patients who use 3 or more tubes of rhPDGF-BB gel [58].

Double-blind, placebo-controlled trials of other growth factors in venous ulcers—epidermal growth factor (EGF) [59], human growth factor [60], granulocyte-macrophage colony-stimulating factor (GMCSF) [61], and transforming growth factor-beta2) [62]—have not demonstrated significant improvements in ulcer healing.

### Lack of Response of Wounds to Topical Growth Factors

This lack of response may be due to a number of factors including the following: degraded ECM; inefficient presentation of growth factors to the cell surface receptors in ways that do not reflect normal in vivo cell surface receptor activation; use of single growth factors instead of more physiologic combinations of growth factors; delivery of free growth factors instead of growth factors bound to or associated with appropriate ECM proteins; and the rapid loss of growth factors from the wound site, possibly due to diffusion or degradation. There is some evidence of mild to complete degradation in venous ulcer wound fluid of proteins such as vitronectin [63] and EGF [64]; therefore, the lack of response to individual topical growth factors for VLUs may be due to the breakdown of the growth factors by proteases in the chronic wound environment [65]. The lack of response of VLU to topical growth factors may be reversed by reducing the protease activity in CWF, and it is postulated that the response of DFU to topical growth factors will be enhanced by reducing protease activity.

### Point-of-Care Testing for Protease Level

A novel point-of-care testing tool, WOUNDCHEK Protease Status (WOUNDCHEK Laboratories), has been developed to determine whether wounds have an increased total protease activity. This test indicates that the total protease activity is either elevated or not elevated based on a predetermined level. It does not give a quantitative measure of total or individual protease levels. If the predetermined levels are meaningful, the test may help to guide the usage of topical agents including growth factors and protease inhibitors. There are 2 registered, ongoing, and unpublished noncomparative studies of the WOUNDCHEK Protease Status test that started recruiting in March 2012, with estimated completions in March 2013 [66], although the results are not as yet available. The studies are designed to enter 250 patients—one study for VLU and another for DFU. Each study aims to determine whether wounds with elevated total protease levels treated with protease modulating therapies have improved clinical healing trajectories at 4 weeks, which are indicative of healing outcomes at 12 weeks. There is a need for randomized controlled trials to determine whether the use of protease inhibitors guided by WOUNDCHEK Protease Status test is accurate and sensitive as compared with using a laboratory protease level assay. If WOUNDCHEK Protease Status test demonstrates clinical utility, it could be used in routine real-time wound management practice.

Recent work in the principal investigator’s laboratory has identified a potential biomarker that with a single test can determine the state of healing of a wound. In this study, GMCSF levels in CWF were elevated in nonhealing compared with healing wounds with apparent separation between the 2 groups. Overall, 90% of nonhealing wounds had a level above an observed threshold. This biomarker test, in combination with a point-of-care protease test such as WOUNDCHEK Protease Status, would provide tools for the clinician to make informed decisions on the use of topical protease inhibitors and growth factors in real time. This would represent a major advance in current wound management and be a completely novel paradigm of therapy for VLU and DFU.

### Novel Approach to Improving Healing

The novel concept of this study is to translate the different elements of research on topical growth factors, protease inhibitors, and point-of-care testing in VLU and DFU into a paradigm of treatment that combines topical growth factors and protease inhibition. This may enable topical growth factors to be effective where they have not been previously in VLU and may enhance the benefit that has been shown in DFU. This model is based on modifying CWF to have more of the characteristics of acute wound fluid. A proof-of-concept study is needed to evaluate whether the use of topical protease inhibitors in both VLUs and DFUs inhibits protease activity and protects topically applied growth factors from degradation and whether this can be monitored by existing or future potential point-of-care tests.

### Hypothesis

A protease inhibitor in combination with PDGF will reduce protease levels and prevent growth factor degradation in chronic VLUs and DFUs as compared with a placebo.

### Primary Aim

The primary aim of this study was to determine whether the topical application of a protease inhibitor in combination with a topical growth factor prevents the degradation of applied growth factors in chronic VLUs and DFUs.

### Secondary Aims

The secondary aims were as follows:

1. To determine whether the topical application of a protease inhibitor in combination with a topical growth factor reduces protease levels in chronic VLUs and DFUs
2. To determine whether the topical application of a protease inhibitor increases the levels of endogenous growth factors in chronic VLUs and DFUs
3. To determine whether there is an increase in healing rates at 4 weeks for both chronic VLUs and chronic DFUs treated with a protease inhibitor dressing in combination with topical PDGF as compared with a placebo
4. To determine the validity of the WOUNDCHEK Protease Status point-of-care test using the gold standard wound fluid assay of total protease
5. To determine whether the changes in protease and growth factor levels are associated with changes in the potential biomarker of wound healing, GMCSF
6. To pilot a cost diary for a possible subsequent phase 3 randomized controlled trial whereby cost-effectiveness will be evaluated

## Methods

### Trial Design

This is a proof-of-concept study that is a double-blind, randomized controlled trial. This trial has received provisional approval from the Hamilton Integrated Research Ethics Board, project number 2067.

### Planned Interventions

Participants will be randomized to receive a protease inhibitor or a placebo for 4 weeks of the study, in combination with a topical growth factor. All participants will receive just the topical growth factor for 2 weeks to obtain baseline data on growth factor and protease levels in the wound fluid.

The interventions patients will receive are as follows:

1. All participants will have Regranex gel (Smith & Nephew) containing a recombinant human PDGF for topical administration, applied to their leg or foot ulcers on a weekly basis for 6 weeks. Regranex is a nonsterile, sodium carboxymethylcellulose-based topical gel. Each gram of Regranex gel contains 100 g of rhPDGF-BB. This is applied by measuring a length of gel from a 15 g tube and is applied at a rate of 0.25 cm length of gel per cm^2^ of ulcer area. A maximum of 2 tubes will be used for each patient, and each patient will have his or her own treatment tubes to ensure that the limit of 2 tubes is not exceeded.
2. Participants randomized to receive the protease inhibitor will receive Promogran (Johnson & Johnson) cut to the size of the ulcer and applied over the rhPDGF-BB.
3. Participants in the placebo arm will receive Aquacel (ConvaTec) dressing applied in a similar manner to the Promogran. This dressing is very similar in appearance to Promogran and has no active protease inhibition function.
4. VLU participants will have an absorbent pad and a 4-layer compression bandage system (Profore, Smith & Nephew) applied over the dressings.
5. Diabetic foot ulcer participants will have an absorbent pad fixed with tape applied over the dressings.

### Randomization Method

Randomization will occur after the first week in which the participant’s compliance with optimal standard therapy is assessed. If the participant is not compliant at this point, he or she will not be randomized into the study. The research coordinator will randomly assign eligible participants using a computer-generated randomization tool. Following eligibility screening by the research coordinator, the system will generate a unique number. The research coordinator then telephones the treatment nursing staff and reports the number. In turn, treatment research nurses refer to a manual of unique numbers generated by an independent biostatistician before study activation to determine the study intervention allocated to the randomized patient. The randomization schedule will be stratified by wound type (VLU or DFU) in variable blocks of 4 and 6. Randomized patients will receive all treatments during the study period according to the intervention they were allocated. The patients, study investigators, research coordinator, laboratory technologists, and assessing research nurses will be blinded to the treatment allocation. Only the treating team members are not blinded.

### Protecting Against Bias

The researchers assessing the protease, growth factor, and biomarker levels in the wound fluid will be blinded to the treatment group, as will the researcher who performs the measurements on the wounds. A dressing similar in appearance to Promogran will be used in the control group so that the participants will also be blinded.

### Inclusion and Exclusion Criteria

Inclusion criteria were as follows:

- Men and women aged ≥18 years
- Ulcer size 1-64 cm^2^
- Ulcer extends through both the epidermis and dermis, with no exposed tendon or bone
- Ulcer duration >3 months and <12 months
- Ulcer located between and including the knee and ankle
- For VLUs—venous refilling time <25 s on photoplethysmography or abnormal venous insufficiency duplex scan
- For DFUs—confirmed type 1 or type 2 diabetes mellitus with a hemoglobin A1C <12%
- Wounds that have not been treated with Promogran in the previous 4 weeks
- Patients able to give informed consent

Exclusion criteria were as follows:

- Ankle-brachial index <0.8
- Ulcer with local or systemic signs of infection
- Patients who have been previously treated with rhPDGF-BB gel
- Patients receiving corticosteroids or immune suppressants
- History of autoimmune disease
- Uncontrolled diabetes (baseline hemoglobin A1C >12%)
- Severe rheumatoid arthritis
- Uncontrolled congestive heart failure
- Malnutrition (albumin <2.5 g/dL)
- Unable to adhere to the protocol
- Known sensitivities to the wound dressings used in the trial
- History of any previous malignancy
- Pregnant or lactating woman

### Treatment Period

Participants will be in the study for a total of 7 weeks. During the first week, participants will not receive the study treatment. During this week, the participants with VLUs will have a standardized bandaging regimen applied and the DFU participants will have their pressure off-loading optimized. Once they have demonstrated tolerance and compliance for wearing these bandaging, they will continue for 6 weeks of study treatment.

### Frequency and Duration of Follow-Up

Patients will be followed up 3 times in a week by a research clinical nurse for the treatment and by a blinded research nurse for outcome measurements (Table 1). The first visit could take up to 1.5 hours as wound fluid collection can be slow. The second visit would be scheduled within a 24-hour period and could take up to 1.5 hours for wound fluid collection. This visit would include the application of the protease inhibitor but not the growth factor. The third visit would require 45 min and it is not measurement visit. Table 1 contains the treatments and measurements at each visit for the each of the treatment weeks. There will be one final visit at the end of the last week of treatment.

At the first follow-up visit each week, the following will be performed:

1. The wound dressing will be taken down and the wound cleansed.
2. The surface area of the wound will be traced and the area will be measured using the Visitrak device (Smith & Nephew).
3. A photograph of the wound will be taken using a standardized protocol.
4. The amount of rhPDGF-BB to be applied for that week will be calculated based on the ulcer size.
5. A swab of the fluid on the surface of the wound will be taken using the WOUNDCHEK system to assess for elevated protease activity.
6. A sample of wound fluid will be collected using a standardized methodology.
7. The rhPDGF-BB will be applied to the wounds.
8. The protease inhibitor dressing or the placebo will be applied.
9. The secondary dressings will be applied.

At the second follow-up visit each week, the following will be performed:

1. Wound fluid will be collected.
2. No measurements will be done; wound photography will be taken.
3. The rhPDGF-BB will be applied to the wounds.
4. The protease inhibitor dressing or the placebo will be applied.
5. The secondary dressings will be applied.

At the third follow-up visit in the same week, the following will be performed:

1. No measurements, wound photography or fluid collection.
2. The rhPDGF-BB will be applied to the wounds.
3. The protease inhibitor dressing or the placebo will be applied.
4. Secondary dressings will be applied.

**Table 1:** Treatments and measurements at each study visit.

Day	Growth factor application	Protease inhibitor application	Wound fluid collection	Point of Careswab for total protease activity (WOUNDCHEK)
1	x	x	x	x
2	x	x	x	x
5	x	x		x

### Outcome Measures

Primary outcome:

- The difference in levels of nondegraded PDGF in CWF from the treatment and placebo treatment groups

Secondary outcomes:

- The difference in levels of total protease and individual proteases in CWF from the treatment and placebo treatment groups as determined by enzyme-linked immunosorbent assay (ELISA) assay
- The difference in levels of endogenous growth factors in CWF from the treatment and placebo treatment groups as determined by ELISA assay
- Percentage reduction in size of ulcers after 1 month of therapy and the proportion of VLU and DFU that are in a healing trajectory. In VLU, a wound area reduction of 40% within 4 weeks is predictive of healing at 12 weeks [67], and in DFUs, a wound area reduction of greater than 50% in 4 weeks is predictive of healing at 12 weeks [68]
- The presence of elevated total protease activity using WOUNDCHEK and the change with time
- The presence of a healing or nonhealing status as measured by GMCSF levels in CWF and the change with time as measured by ELISA assay
- The completeness, ease of use, literacy, and timeliness of completing the pilot cost diary from a societal perspective. Both medical staff and participants will complete cost diaries

### Measurement of Outcomes at Follow-Up Visits

At the first follow-up visit each week, the following will be performed:

1. The surface area of the wound will be traced and the area will be measured using the Visitrak device (Smith & Nephew).
2. A photograph of the wound will be taken using a standardized protocol.
3. A swab of the fluid on the surface of the wound will be taken using the WOUNDCHEK system to assess for elevated protease activity. The swabs will be stored for subsequent analysis of actual protease levels.
4. A sample of wound fluid will be collected from the chronic wounds by covering the wound with a transparent occlusive dressing (Opsite, Smith & Nephew, Hull, UK) for approximately 1 hour. Fluid that has accumulated will then be aspirated and stored at −80°C. The wound fluid samples will be aliquoted into 20 ul samples and will be stored at −80°C. At the completion of the study, the samples will be analyzed using Quantibody analysis to measure the levels of proteases and nondegraded growth factors in the wound fluid, and a Western blot will be performed to assess rhPDGF-BB degradation. All of these samples will be assayed at the end of the study.

At the second visit in the same week, the following will be performed:

1. A sample of wound fluid will be collected.
2. A swab of the fluid on the surface of the wound will be taken using the WOUNDCHEK system to assess for elevated protease activity.

At the third follow-up visit in the same week, the following will be performed:

1. Swab of the fluid on the surface of the wound will be taken using the WOUNDCHEK system to assess for elevated protease activity.
2. The cost diary for that week will be completed.

### Health Services Issues

As this is a proof-of-concept study of the biochemical efficacy of the therapy, no health service evaluations will be undertaken; however, a cost diary from a societal perspective will be validated in preparation for a future study.

### Sample Size

One small study has assessed the levels of proteases in CWF from VLU after the application of Promogran [43]. In that study, the reduction in elastase after 5 days was approximately 80%, and the reduction in MMP-9 was approximately 45%. There are no data on the degradation of rhPDGF-BB that is applied topically to VLU or DFU. For the sample size analysis, a modest reduction in protease levels of 25% was estimated. Due to the variability of factors, an SD of 35% of the value for the control levels was used. Using a power of 80% and a 5% level of significance, the calculated sample size was 31 in each group. Due to possible nonadherence with the study and collection of samples, the number of participants to be recruited is 40 in each group.

### Planned Recruitment Rate

Patients will be approached and recruited by the research coordinator after being identified in the clinic by the participating physicians. The study will be conducted over 3 years. The first 3 months requires study preparation including intensive protocol training to ensure reliability. A number of protocols such as wound fluid collection are not typical wound care procedures. On the basis of previous studies from this laboratory, recruitment will need 2 years, given the average of 3.5 participants recruited per month. The last 3 months will include conducting the assay analysis and data analysis and disseminating the results.

### Compliance

On the basis of previous studies from this laboratory, compliance is a risk, given the complexity of the participants’ chronic wound, comorbidities, and tolerance for either compression bandaging or pressure off-loading. Compliance will be assessed on the participants’ second visit by noting if they are wearing their prescribed compression bandaging or pressure off-loading device. If they are not complying with standard therapy, the participants will not enter the treatment segment of the study. The need to attend the study clinic 3 times a week for 6 weeks is demanding and risks noncompliance; however, these visits replace the need for the participants to attend their regular clinic to have their dressings changed.

### Likely Rate of Loss to Follow-Up

An allowance has been made for just over 20% loss of participants to follow-up. To assist participants in attending the visits, reimbursement of costs for travel and parking will be offered in the form of gift certificates. The average of these would be Can $40.00.

### Number of Centers

A total of 2 sites within the same health region are required to meet the recruitment targets.

### Data Analysis

Data analysis will be performed on the outcome measures as follows:

1. The trial will meet Consolidated Standards of Reporting trials (CONSORT) requirements for reporting of randomized controlled trials. Baseline characteristics of patients in the 2 treatment groups will be reported using frequency distributions and descriptive statistics including measures of central tendency and dispersion.
2. The changes in levels of nondegraded PDGF in CWF from the treatment and placebo treatment groups will be analyzed using repeated measures analysis of variance (ANOVA), using the wound type and treatment group as covariates.
3. The changes in levels of proteases and endogenous growth factors in CWF from the treatment and placebo treatment groups will be analyzed using repeated measures ANOVA, with the wound type and treatment group as covariates.
4. The percentage change in wound area after 4 weeks in the study will be assessed on an intention-to-treat basis using Student *t* test. On the basis of the percentage reduction in size at 4 weeks, the ulcers will be categorized as predicted to heal or not heal at 12 weeks. These healing predictions for the Promogran and placebo groups will be assessed by chi-squared analysis.
5. The change in total protease status after application of the Promogran or the placebo will be assessed by chi-squared analysis.
6. The change in healing status as determined by GMCSF biomarker after application of the Promogran or the placebo will be assessed by chi-squared analysis. Healing analysis will be performed on an intention-to-treat basis.
7. All analysis will be completed using SPSS version 22 (IBM Corporation, Armonk, New York).

### Frequency of Analyses

All statistical analyses will be performed only at the completion of the study and after final data checking is completed.

### Planned Subgroup Analyses

If the initial analysis demonstrates a difference between VLU and DFU responses to protease inhibitor application, subgroup analyses of these 2 groups will be performed.

### Previous Pilot Testing

There is one small study that has demonstrated reduction in elastase and MMP-9 levels in CWF from VLU with the application of Promogran [43]. There is one study that has demonstrated degradation of EGF by CWF, but none that has demonstrated degradation of PDGF in VLU or DFU [26].

### Trial Management

The study will employ a research coordinator 2 days per week to manage data handling, randomization, and other aspects of the study. A total of 2 clinical research nurses will be employed to perform the dressings and application of the study treatments.

The principal applicant will oversee, in conjunction with the research coordinator, the management of the study, the location and facilities for the study, treatment materials and data storage systems, and the analysis of the data from the study.

A study steering committee will be formed from experienced researchers within the Population Health Research Institute in Hamilton. A data and safety monitoring board will be blinded to treatment group. The board will review serious adverse events.

## Results

The project was funded by the Canadian Institutes for Health Research and enrollment is expected to be initiated in 2018. It is expected that results will be available in 2021.

## Discussion

It is expected that the results of this trial will inform as to whether modifying the wound environment through the use of protease inhibitors increases the effectiveness of topically applied growth factors in the healing of chronic wounds.

## Abbreviations

ANOVA: analysis of variance
CWF: chronic wound fluid
DFU: diabetic foot ulcer
ECM: extracellular matrix
EGF: epidermal growth factor
ELISA: enzyme-linked immunosorbent assay
GMCSF: granulocyte-macrophage colony-stimulating factor
MMP: matrix metalloprotease
PDGF: platelet-derived growth factor
rhPDGF-BB: recombinant human PDGF-BB
TIMP: tissue inhibitors of metalloproteinase
VLU: venous leg ulcer
